# Crystal structure and Hirshfeld surface analysis, crystal voids, inter­action energy calculations and energy frameworks of *C*-anthracen-9-yl-*N*-methyl aldo­nitrone

**DOI:** 10.1107/S2056989026000599

**Published:** 2026-01-29

**Authors:** Jamal Lasri, Mohamed M. Zayed, Yaseen A. Almehmadi, Naser E. Eltayeb, Tuncer Hökelek, Aidan P. McKay

**Affiliations:** a Department of Chemistry Rabigh College of Science and Arts King Abdulaziz University,Jeddah 21589 Saudi Arabia; b Environmental and Occupational Medicine Department, National Research Centre, Giza 12622, Egypt; c King Fahd Medical Research Center King Abdulaziz University,Jeddah 21589 Saudi Arabia; d Department of Chemistry, Faculty of Pure and Applied Sciences, International University of Africa, Khartoum 2469, Sudan; e Department of Physics, Hacettepe University, 06800 Beytepe, Ankara, Türkiye; f EaStCHEM School of Chemistry, University of St Andrews, Fife KY16 9ST, United Kingdom; Katholieke Universiteit Leuven, Belgium

**Keywords:** *C*-anthracen-9-yl-*N*-methyl aldo­nitrone, crystal structure, hy­dro­gen bond, π-stacking, Hirshfeld surface, energy framework analysis

## Abstract

The title com­pound (systematic name: 1-anthracen-9-yl-*N*-methyl­methanimine oxide), C_16_H_13_NO, contains an almost planar anthracene ring system [r.m.s. deviation = 0.021 (1) Å]. In the crystal, inter­molecular bifurcated C—H⋯O hy­dro­gen bonds link the mol­ecules into infinite chains along the *a*-axis direction. The π–π stacking inter­actions between the benzene rings of adjacent mol­ecules help to consolidate the three-dimensional architecture. The Hirshfeld surface analysis of the crystal structure indicates that the most important contributions for the crystal packing are from H⋯H (54.5%), H⋯C/C⋯H (23.7%), H⋯O/O⋯H (10.6%) and C⋯C (9.8%) inter­actions. The volume of the crystal voids and the percentage of free space were calculated to be 76.07 Å^3^ and 6.57%, respectively, showing that there is no large cavity in the crystal packing. Evaluation of the electrostatic, dispersion and total energy frameworks indicates that the stabilization largely depends on dispersion energy contributions. Hydrogen bonding, π–π and van der Waals inter­actions, together with the dispersion energy contributions, are the dominant inter­actions in the crystal packing.

## Abstract 2

The title com­pound contains an almost planar anthracene ring system. In the crystal, inter­molecular bifurcated C—H⋯O hy­dro­gen bonds link the mol­ecules into infinite chains along the *a*-axis direction and π–π stacking inter­actions between the benzene rings of adjacent mol­ecules help to consolidate the three-dimensional architecture.

## 1. Chemical context

Nitro­nes have inter­esting applications as buiding blocks in the synthesis of natural products (Padwa & Pearson, 2002 ▸) and have found usage as both modifiers in radical polymerization and regulators of mol­ecular weight (Feuer, 2007 ▸; Hamer & Macaluso, 1964 ▸). Nitro­nes have been broadly used in metal-mediated [2 + 3]-cyclo­addition reactions to furnish N-heterocyclic com­pounds which have shown to be excellent catalysts for Suzuki-Miyaura C—C cross-couplings (Fer­nan­des *et al.*, 2011 ▸). Nitro­nes have also been used for therapeutic applications as they are components of the mol­ecular structure of several drugs (Floyd *et al.*, 2008 ▸). Currently, our research program focuses on the synthesis, X-ray structure analysis, Hirshfeld surface analysis and density functional theory (DFT) calculations and mol­ecular docking studies of aldo­nitrone-type com­pounds (Lasri *et al.*, 2024 ▸). Herein, we report the synthesis, mol­ecular and crystal structures, Hirshfeld surface analysis, crystal voids, inter­action energies and energy frameworks of the title com­pound *C*-anthracen-9-yl-*N*-methyl aldo­nitrone, (I).
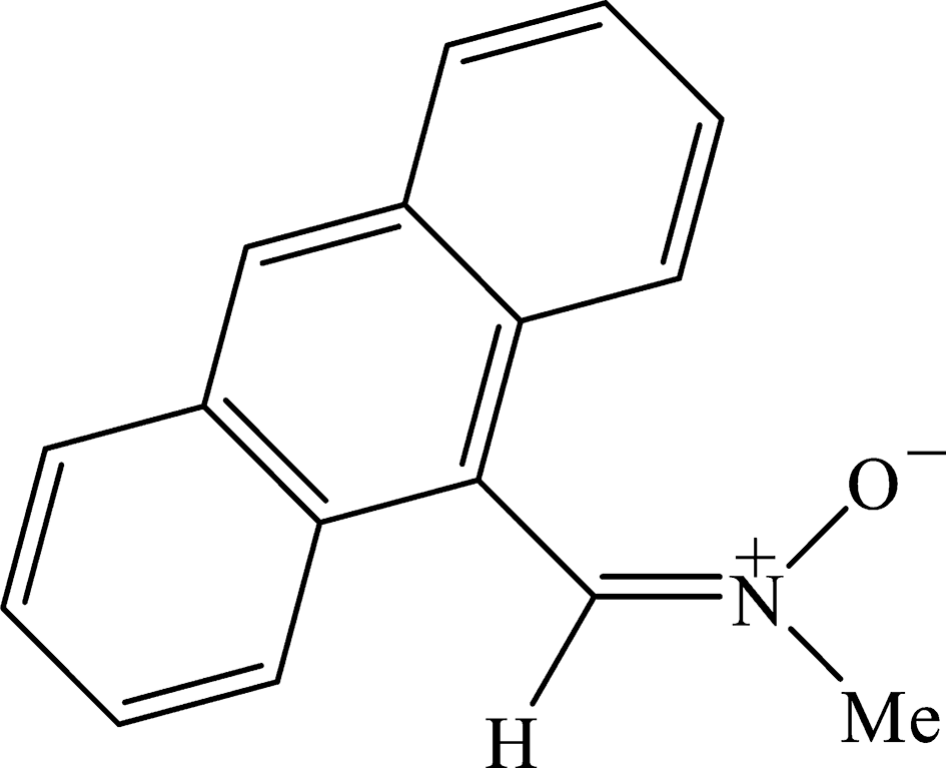


## 2. Structural commentary

**Figure 1:**
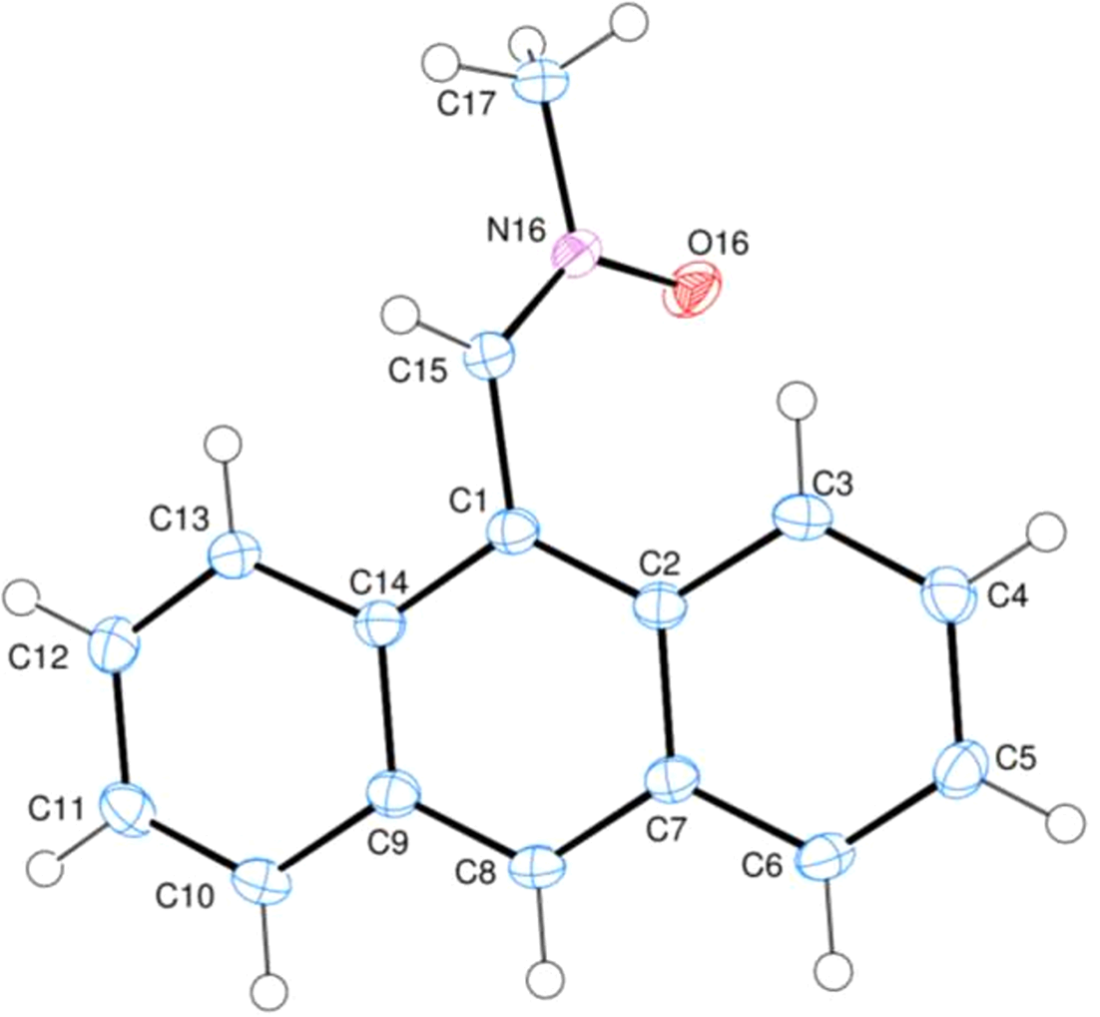
The mol­ecular structure of the title mol­ecule with the atom-numbering scheme and 50% probability displacement ellipsoids.

The title com­pound, (I), contains an almost planar [r.m.s. deviation = 0.021 (1) Å] anthracene ring system, consisting of three fused benzene rings, denoted *A* (C1/C2/C7–C9/C14), *B* (C2–C7) and *C* (C9–C14) (Fig. 1 ▸). Atom C3 deviates by −0.0419 (14) Å from the least-squares plane through the ring system. The planes of benzene rings *A*, *B* and *C* are oriented at dihedral angles of *A*/*B* = 1.95 (4)°, *A*/*C* = 1.99 (5)° and *B*/*C* = 0.51 (3)°. In the substituent, the C1—C15—N16, C15—N16—C17, C15—N16—O16 and C17—N16—O16 bond angles are 125.01 (13), 119.73 (12), 125.30 (12) and 114.96 (11)°, respectively. On the other hand, the N16—C15—C1—C2, N16—C15—C1—C14, C15—C1—C2—C3 and C15—C1—C14—C13 torsion angles are 56.39 (19), −128.50 (14), −1.4 (2) and 4.96 (19)°, respectively. The dihedral angle between the plane of the anthracene ring and the least-squares plane through its substituent is 54.42 (5)°.

## 3. Supra­molecular features

**Figure 2:**
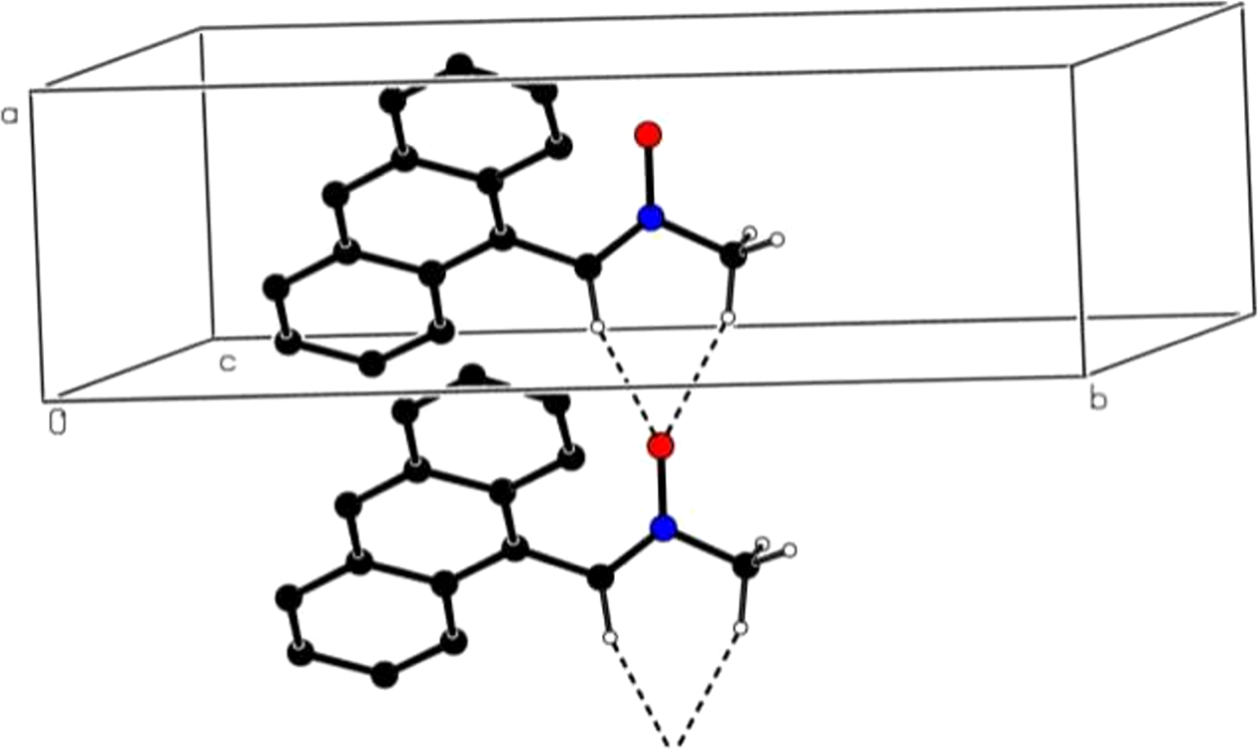
A partial packing diagram viewed down the *c*-axis direction. Inter­molecular C—H⋯O hy­dro­gen bonds are shown as dashed lines. Nonbonding H atoms have been omitted for clarity.

In the crystal, inter­molecular bifurcated C—H⋯O hy­dro­gen bonds (Table 1 ▸) link the mol­ecules into infinite chains along the *a*-axis direction (Fig. 2 ▸). The π–π stacking inter­actions between the benzene rings (*A*, *B* and *C*) of adjacent mol­ecules, with inter-centroid distances of 3.8445 (8) [between rings *A* and *B*, α = 1.96 (6)° and slippage = 1.592 Å] and 3.8497 (8) Å [between rings *A* and *C*, α = 0.51 (7)° and slippage = 1.636 Å] may help to consolidate the three-dimensional architecture, together with C—H⋯O contacts. No C—H⋯π(ring) inter­actions are identified.

**Figure 3:**
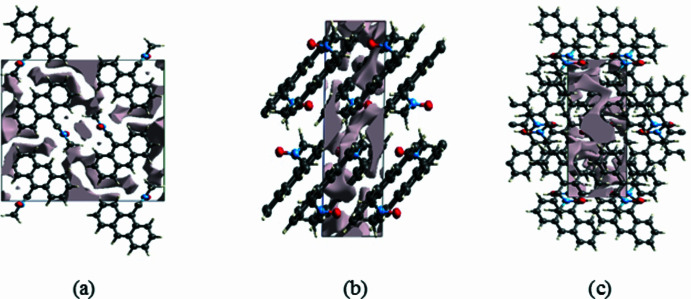
Graphical views of voids in the crystal packing of the title com­pound (*a*) along the *a*-axis, (*b*) along the *b*-axis and (*c*) along the *c*-axis direction.

A void analysis was performed by adding up the electron densities of the spherically symmetric atoms contained in the asymmetric unit (Turner *et al.*, 2011 ▸). The volume of the crystal voids [Figs. 3 ▸(*a*), 3(*b*) and 3(*c*)] and the percentage of free space in the unit cell are calculated as 76.07 Å^3^ and 6.57%, respectively, indicating that the crystal packing is com­pact.

## 4. Hirshfeld surface analysis

**Figure 4:**
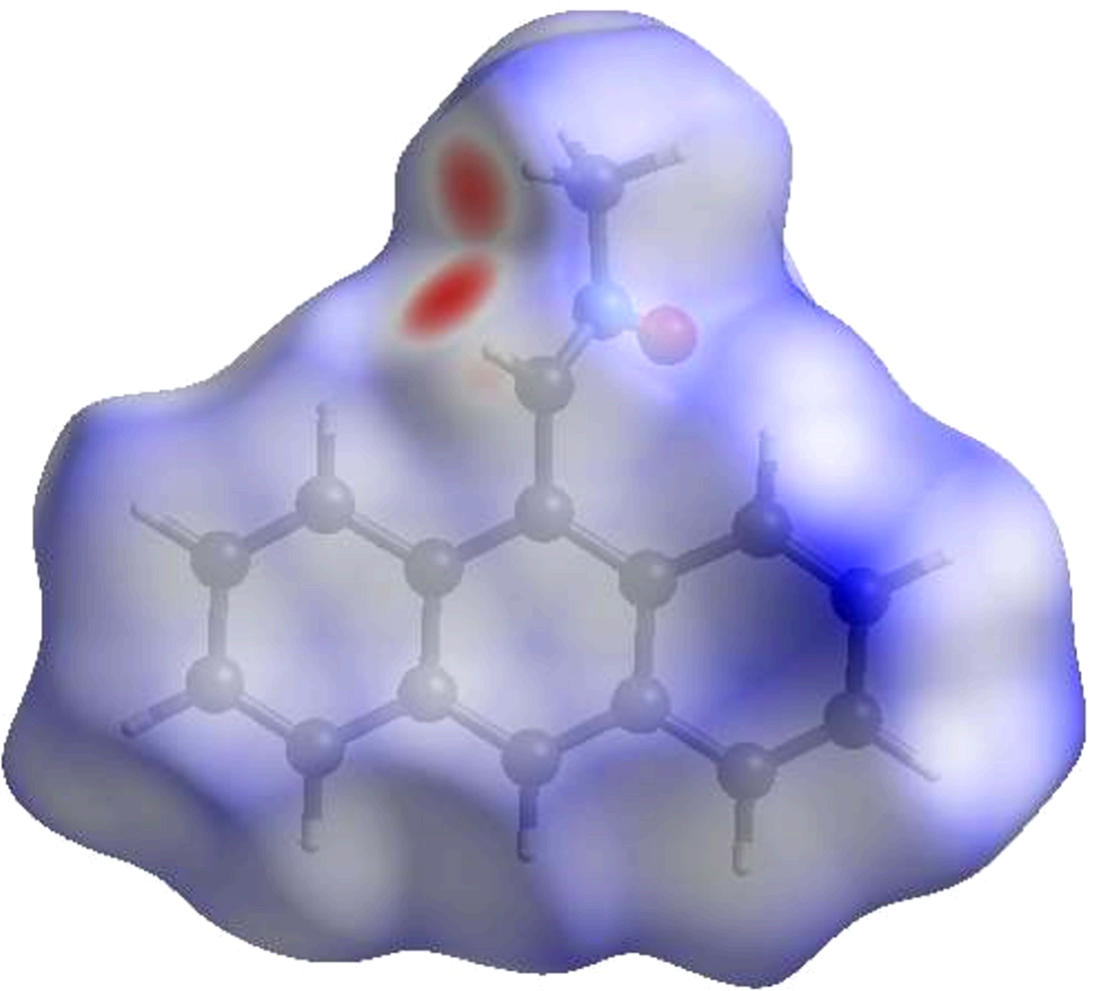
View of the three-dimensional Hirshfeld surface of the title com­pound plotted over *d*_norm_ in the range from −0.3681 to 1.4279 a.u.

**Figure 5:**
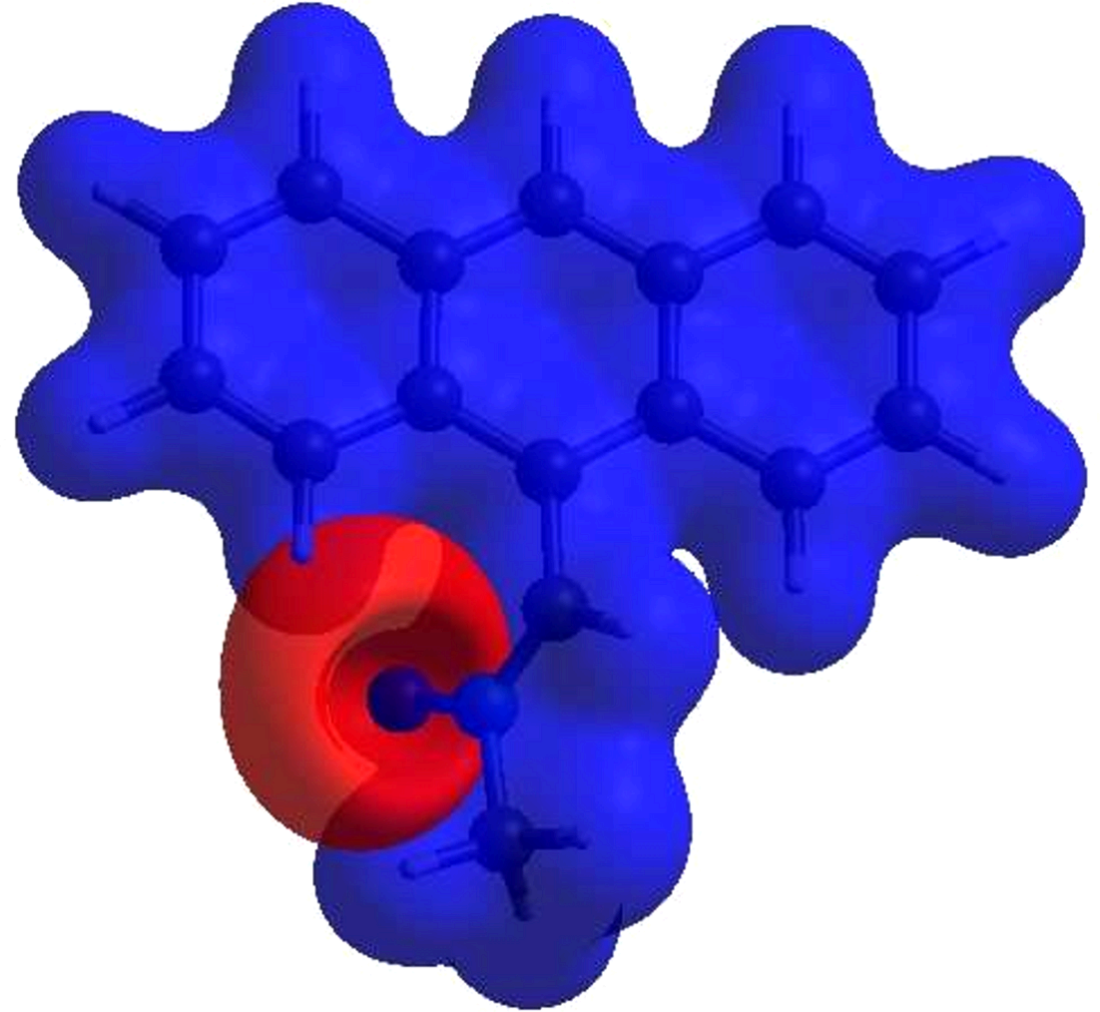
View of the Hirshfeld surface of the title com­pound plotted over electrostatic potential energy in the range from −0.0500 to 0.0500 a.u. using the STO-3G basis set at the Hartree–Fock level of theory. Hydrogen-bond donors and acceptors are shown as blue and red regions around the atoms corresponding to positive and negative potentials, respectively.

**Figure 6:**
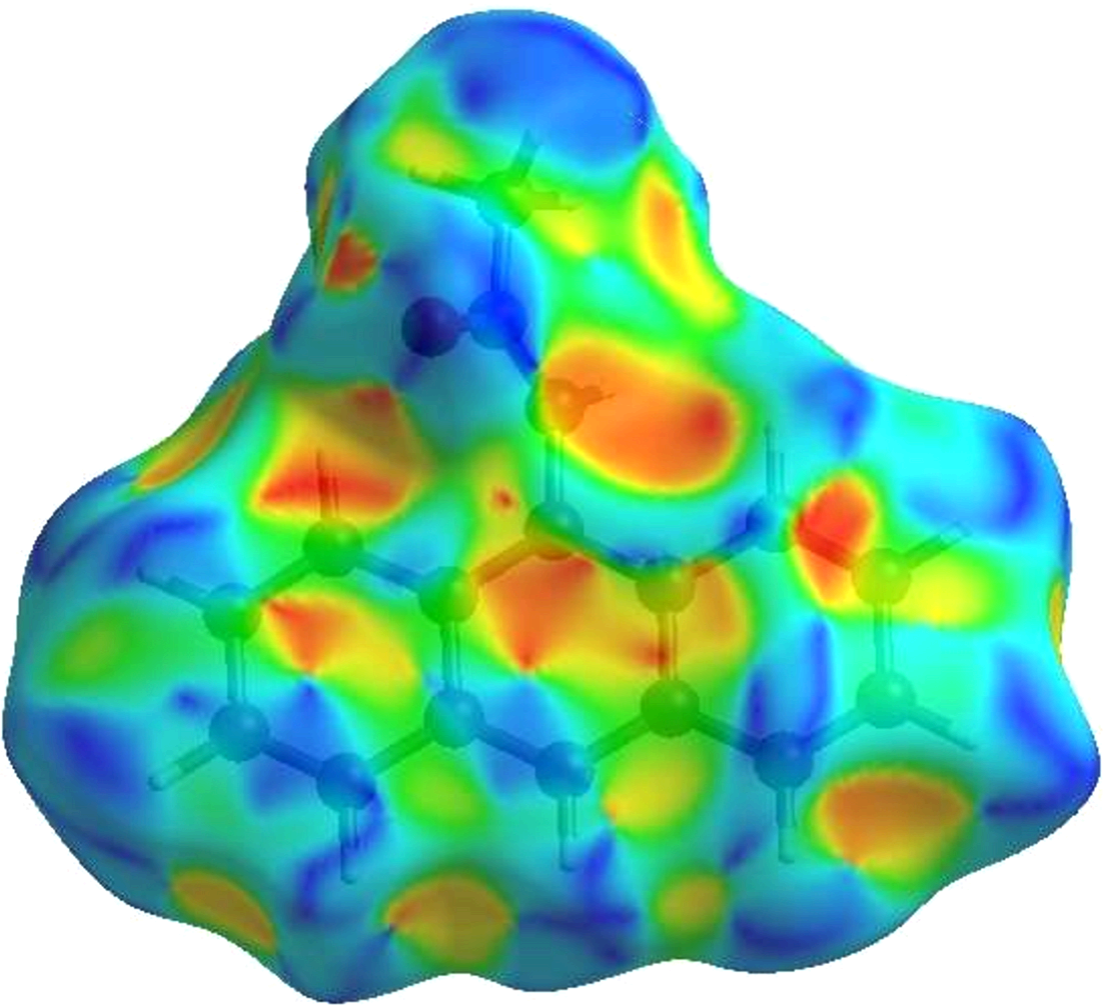
Hirshfeld surface of the title com­pound plotted over shape-index.

**Figure 7:**
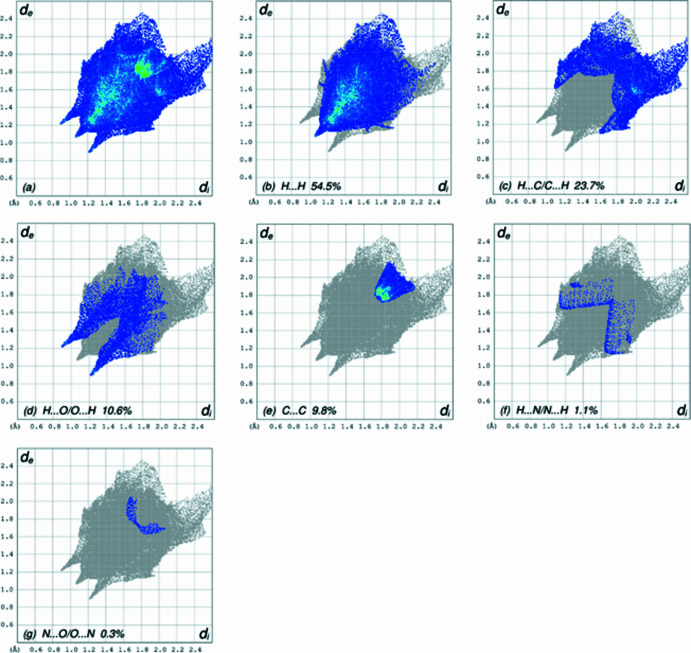
The full two-dimensional fingerprint plots for the title com­pound, showing (*a*) all inter­actions, and delineated into (*b*) H⋯H, (*c*) H⋯C/C⋯H, (*d*) H⋯O/O⋯H, (*e*) C⋯C, (*f*) H⋯N/N⋯H and (*g*) N⋯O/O⋯N inter­actions. The *d*_i_ and *d*_e_ values are the closest inter­nal and external distances (in Å) from given points on the Hirshfeld surface contacts.

A Hirshfeld surface (HS) analysis was carried out using *CrystalExplorer* (Version 17.5; Spackman *et al.*, 2021 ▸) for clarifying the inter­molecular inter­actions in the crystal of (I). The HS plotted over *d*_norm_ is shown in Fig. 4 ▸, where the bright-red spots correspond to donor and/or acceptor sites; they also appear as blue and red regions in Fig. 5 ▸, corresponding to positive and negative potentials (Spackman *et al.*, 2008 ▸). The shape-index surface can be used for identifying the characteristic packing modes, particularly, the pres­ence of aromatic stacking inter­actions like C—H⋯π(ring) and π–π inter­actions, with the former represented as red π-holes, which are related to the electron–ring inter­actions between the C—H groups with the centroids of the aromatic rings of neighbouring mol­ecules. Fig. 6 ▸ clearly suggests that there are no C—H⋯π(ring) inter­actions. A π–π stacking is indicated by the presence of adjacent red and blue triangles, as clearly indicated by Fig. 6 ▸. According to the 2D fingerprint plots (McKinnon *et al.*, 2007 ▸), inter­molecular H⋯H, H⋯C/C⋯H, H⋯O/O⋯H and C⋯C contacts make important contributions to the HS, with values of 54.5, 23.7, 10.6 and 9.8%, respectively (Fig. 7 ▸).

## 5. Database survey

A survey of the Cambridge Structural Database (CSD, Version 6.01, November 2025 update; Groom *et al.*, 2016 ▸) revealed 3581 C9-substituted anthracene derivatives. Herein, we present 20 of them with structural similarity to the target com­pound *C*-anthracen-9-yl-*N*-methyl aldo­nitrone, namely, **I** (AWUZOI; Kraicheva *et al.*, 2011 ▸), **II** (AXODAS; Wong *et al.*, 2004 ▸), **III** (AZORUD; Geetha *et al.*, 2011 ▸), **IV** (CEJLOT; Horiguchi & Ito, 2006 ▸), **V** (CUBMOC; Jaworska *et al.*, 2009 ▸), **VI** (EDOHIS; Monika *et al.*, 2022 ▸), **VII** (FAXVIK; Howie *et al.*, 2005 ▸), **VIII** (FIBQIT; Spinelli *et al.*, 2018 ▸), **IX** (FOHLOE; Howie & Wardell, 2005 ▸), **X** (GUMLAD; Lohar *et al.*, 2015 ▸), **XI** (KEYJAD; Kakimoto *et al.*, 2023 ▸), **XII** (KOBWAC; Ghosh *et al.*, 2017 ▸), **XIII** (NIJWEK; Banerjee *et al.*, 2013 ▸), **XIV** (NOKMIN; Zheng *et al.*, 2024 ▸), **XV** (OCOLUQ; Faizi *et al.*, 2017 ▸), **XVI** (PIGWOR; Subramanian *et al.*, 1993 ▸), **XVII** (QARBUG; Ihmels *et al.*, 2000 ▸), **XVIII** (TITNES; Junor *et al.*, 2019 ▸), **XIX** (TUPGIV; Villalpando *et al.*, 2010 ▸) and **XX** (YIVQAY; Barwiolek *et al.*, 2019 ▸).

## 6. Inter­action energy calculations and energy frameworks

**Figure 8:**
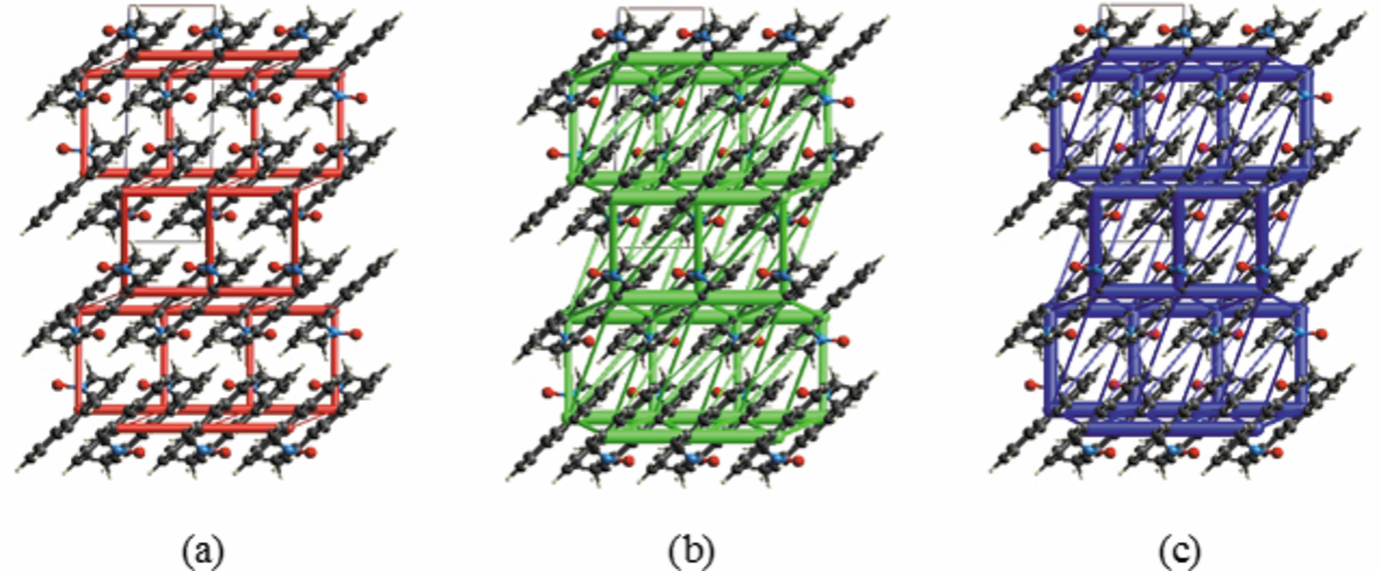
The energy frameworks for a cluster of mol­ecules of the title com­pound viewed down the *b* axis, showing the (*a*) electrostatic energy *E*_ele_, (*b*) dispersion energy *E*_dis_ and (*c*) total energy *E*_tot_ diagrams. The cylindrical radius is proportional to the relative strength of the corresponding energies and they were adjusted to the same scale factor of 80 with a cut-off value of 5 kJ mol^−1^ within 2×2×2 unit cells.

The CE-B3LYP/6-31G(d,p) energy model available in *CrystalExplorer* (Version 17.5; Spackman *et al.*, 2021 ▸) was used to calculate the inter­molecular inter­action energies. Hydrogen-bonding inter­action energies (in kJ mol^−1^) were calculated to be −33.9 (*E*_ele_), −10.9 (*E*_pol_), −62.7 (*E*_dis_), 65.8 (*E*_rep_) and −57.9 (*E*_tot_) for the C15—H15⋯O16, and −23.9 (*E*_ele_), −6.4 (*E*_pol_), −32.8 (*E*_dis_), 18.5 (*E*_rep_) and −47.1 (*E*_tot_) for the C17—H17*B*⋯O16 hy­dro­gen-bond inter­action. Energy frameworks combine the calculation of inter­molecular inter­action energies with a graphical representation of their magnitude (Turner *et al.*, 2015 ▸). Energy frameworks were constructed for *E*_ele_ (red cylinders), *E*_dis_ (green cylinders) and *E*_tot_ (blue cylinders) [Figs. 8 ▸(*a*), 8(*b*) and 8(*c*)], and their evaluation indicates that the stabilization largely depends on dispersion energy contributions in the crystal structure of (I).

## 7. Synthesis and crystallization

To a solution of *N*-methyl­hydroxyl­amine (99.9 mg, 1.20 mmol) in MeOH (50 ml) was added sodium carbonate (63.4 mg, 0.60 mmol) and the reaction mixture was stirred for 10 min followed by the addition of anthracene-9-carbaldehyde (224.4 mg, 1.09 mmol). The mixture was then stirred for 12 h at room tem­per­a­ture. The precipitate which formed was filtered off and MeOH was eliminated *in vacuo*. In order to remove the NaCl produced, the obtained solid was dissolved in CH_2_Cl_2_ and filtered, the filtrate was then evaporated *in vacuo*. The solid product was ultimately washed with Et_2_O to give pure *C*-anthracen-9-yl-*N*-methyl aldo­nitrone, (I). Yellow crystals suitable for X-ray analysis were obtained by slow evaporation of a CH_2_Cl_2_ solution (yield 90%). FT–IR (cm^−1^): 1637 (C=N), 1566 (C=C). Analysis calculated (%) for C_16_H_13_NO: C 81.68, H 5.57, N 5.95; found: C 81.73, H 5.60, N 5.93.

## 8. Refinement

Crystal data, data collection and structure refinement details are summarized in Table 2 ▸. The C-bound H-atom positions were calculated geometrically at distances of 0.95 (for aromatic and methine CH) and 0.98 Å (for CH_3_), and refined using a riding model by applying the constraints *U*_iso_(H) = *kU*_eq_(C), where *k* = 1.5 for CH_3_ and 1.2 for the other H atoms.

## Supplementary Material

Crystal structure: contains datablock(s) I. DOI: 10.1107/S2056989026000599/vm2323sup1.cif

Structure factors: contains datablock(s) I. DOI: 10.1107/S2056989026000599/vm2323Isup2.hkl

checkcif. DOI: 10.1107/S2056989026000599/vm2323sup3.pdf

checkcif. DOI: 10.1107/S2056989026000599/vm2323sup4.pdf

Supporting information file. DOI: 10.1107/S2056989026000599/vm2323Isup5.cml

CCDC reference: 2524759

Additional supporting information:  crystallographic information; 3D view; checkCIF report

## Figures and Tables

**Table 1:** Hydrogen-bond geometry (Å, °)

*D*—H⋯*A*	*D*—H	H⋯*A*	*D*⋯*A*	*D*—H⋯*A*
C15—H15⋯O16^i^	0.95	2.24	3.1267 (18)	154
C17—H17*B*⋯O16^i^	0.98	2.28	3.2037 (18)	157

Symmetry code: (i) 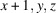.

**Table 2:** Experimental details

Crystal data	
Chemical formula	C_16_H_13_NO
*M*_r_	235.27
Crystal system, space group	Monoclinic, *P*2_1_/*n*
Temperature (K)	100
*a*, *b*, *c* (Å)	4.89615 (14), 16.6590 (5), 14.2008 (4)
β (°)	91.240 (3)
*V* (Å^3^)	1158.02 (6)
*Z*	4
Radiation type	Mo *K*α
μ (mm^−1^)	0.08
Crystal size (mm)	0.26 × 0.02 × 0.01

Data collection	
Diffractometer	Rigaku XtaLAB P200K
Absorption correction	Multi-scan (*CrysAlis PRO*; Rigaku OD, 2024 ▸)
*T*_min_, *T*_max_	0.714, 1.000
No. of measured, independent and observed [*I* > 2σ(*I*)] reflections	24788, 2788, 1992
*R*_int_	0.055
(sin θ/λ)_max_ (Å^−1^)	0.682

Refinement	
*R*[*F*^2^ > 2σ(*F*^2^)], *wR*(*F*^2^), *S*	0.043, 0.124, 1.05
No. of reflections	2788
No. of parameters	164
H-atom treatment	H-atom parameters constrained
Δρ_max_, Δρ_min_ (e Å^−3^)	0.30, −0.20

Computer programs: *CrysAlis PRO* (Rigaku OD, 2024 ▸), *SHELXT2018* (Sheldrick, 2015*a* ▸), *SHELXL2019* (Sheldrick, 2015*b* ▸) and *OLEX2* (Dolomanov *et al.*, 2009 ▸).

## supplementary crystallographic information

### 1-Anthracen-9-yl-N-methylmethanimine oxide Crystal data

**Table:**

C_16_H_13_NO	*F*(000) = 496
*M**_r_* = 235.27	*D*_x_ = 1.349 Mg m^−^^3^
Monoclinic, *P*2_1_/*n*	Mo *K*α radiation, λ = 0.71073 Å
*a* = 4.89615 (14) Å	Cell parameters from 8143 reflections
*b* = 16.6590 (5) Å	θ = 2.8–29.1°
*c* = 14.2008 (4) Å	µ = 0.08 mm^−^^1^
β = 91.240 (3)°	*T* = 100 K
*V* = 1158.02 (6) Å^3^	Platy-needle, yellow
*Z* = 4	0.26 × 0.02 × 0.01 mm

### 1-Anthracen-9-yl-N-methylmethanimine oxide Data collection

**Table:**

Rigaku XtaLAB P200K diffractometer	2788 independent reflections
Radiation source: Rotating Anode, Rigaku FR-X	1992 reflections with *I* > 2σ(*I*)
Rigaku Osmic Confocal Optical System monochromator	*R*_int_ = 0.055
Detector resolution: 5.8140 pixels mm^-1^	θ_max_ = 29.0°, θ_min_ = 1.9°
shutterless scans	*h* = −6→6
Absorption correction: multi-scan (CrysAlis PRO; Rigaku OD, 2024)	*k* = −21→21
*T*_min_ = 0.714, *T*_max_ = 1.000	*l* = −19→19
24788 measured reflections	

### 1-Anthracen-9-yl-N-methylmethanimine oxide Refinement

**Table:**

Refinement on *F*^2^	Primary atom site location: dual
Least-squares matrix: full	Hydrogen site location: inferred from neighbouring sites
*R*[*F*^2^ > 2σ(*F*^2^)] = 0.043	H-atom parameters constrained
*wR*(*F*^2^) = 0.124	*w* = 1/[σ^2^(*F*_o_^2^) + (0.0587*P*)^2^ + 0.3266*P*] where *P* = (*F*_o_^2^ + 2*F*_c_^2^)/3
*S* = 1.05	(Δ/σ)_max_ < 0.001
2788 reflections	Δρ_max_ = 0.30 e Å^−^^3^
164 parameters	Δρ_min_ = −0.20 e Å^−^^3^
0 restraints	

### 1-Anthracen-9-yl-N-methylmethanimine oxide Special details

**Table:**

Geometry. All esds (except the esd in the dihedral angle between two l.s. planes) are estimated using the full covariance matrix. The cell esds are taken into account individually in the estimation of esds in distances, angles and torsion angles; correlations between esds in cell parameters are only used when they are defined by crystal symmetry. An approximate (isotropic) treatment of cell esds is used for estimating esds involving l.s. planes.

### 1-Anthracen-9-yl-N-methylmethanimine oxide Fractional atomic coordinates and isotropic or equivalent isotropic displacement parameters (Å^2^)

**Table:**

	*x*	*y*	*z*	*U*_iso_*/*U*_eq_	
O16	0.2639 (2)	0.47437 (6)	0.60736 (7)	0.0281 (3)	
N16	0.5270 (2)	0.47324 (7)	0.61726 (8)	0.0205 (3)	
C1	0.5642 (3)	0.60243 (8)	0.70066 (9)	0.0178 (3)	
C2	0.3685 (3)	0.60047 (8)	0.77210 (9)	0.0175 (3)	
C3	0.2557 (3)	0.52728 (8)	0.80734 (9)	0.0204 (3)	
H3	0.315933	0.477410	0.782852	0.025*	
C4	0.0635 (3)	0.52796 (8)	0.87536 (9)	0.0229 (3)	
H4	−0.009682	0.478649	0.897013	0.028*	
C5	−0.0289 (3)	0.60146 (9)	0.91436 (9)	0.0232 (3)	
H5	−0.166736	0.601175	0.960406	0.028*	
C6	0.0801 (3)	0.67197 (8)	0.88573 (9)	0.0215 (3)	
H6	0.021220	0.720677	0.913543	0.026*	
C7	0.2818 (3)	0.67444 (8)	0.81452 (9)	0.0183 (3)	
C8	0.3977 (3)	0.74681 (8)	0.78584 (9)	0.0192 (3)	
H8	0.341342	0.795398	0.814553	0.023*	
C9	0.5936 (3)	0.74962 (8)	0.71628 (9)	0.0186 (3)	
C10	0.7105 (3)	0.82395 (8)	0.68640 (10)	0.0222 (3)	
H10	0.655215	0.872636	0.715162	0.027*	
C11	0.8991 (3)	0.82603 (8)	0.61756 (10)	0.0244 (3)	
H11	0.973665	0.875937	0.598406	0.029*	
C12	0.9852 (3)	0.75359 (9)	0.57416 (10)	0.0243 (3)	
H12	1.118288	0.755476	0.526533	0.029*	
C13	0.8788 (3)	0.68159 (8)	0.60015 (9)	0.0211 (3)	
H13	0.937397	0.633996	0.569781	0.025*	
C14	0.6800 (3)	0.67635 (8)	0.67244 (9)	0.0178 (3)	
C15	0.6736 (3)	0.52877 (8)	0.65776 (9)	0.0196 (3)	
H15	0.866041	0.521447	0.659962	0.024*	
C17	0.6620 (3)	0.40225 (8)	0.57657 (10)	0.0228 (3)	
H17A	0.608897	0.354042	0.611222	0.034*	
H17B	0.860711	0.408976	0.581280	0.034*	
H17C	0.606005	0.396676	0.510219	0.034*	

### 1-Anthracen-9-yl-N-methylmethanimine oxide Atomic displacement parameters (Å^2^)

**Table:**

	*U*^11^	*U*^22^	*U*^33^	*U*^12^	*U*^13^	*U*^23^
O16	0.0163 (6)	0.0333 (6)	0.0348 (6)	0.0009 (4)	0.0017 (4)	−0.0111 (5)
N16	0.0171 (6)	0.0207 (6)	0.0237 (6)	0.0009 (5)	0.0036 (5)	−0.0040 (5)
C1	0.0172 (7)	0.0170 (7)	0.0191 (6)	0.0021 (5)	−0.0013 (5)	−0.0019 (5)
C2	0.0184 (7)	0.0162 (6)	0.0178 (6)	0.0020 (5)	−0.0011 (5)	−0.0002 (5)
C3	0.0243 (8)	0.0153 (6)	0.0216 (7)	0.0028 (5)	−0.0002 (6)	−0.0013 (5)
C4	0.0286 (8)	0.0201 (7)	0.0202 (7)	−0.0024 (6)	0.0030 (6)	0.0020 (5)
C5	0.0246 (8)	0.0259 (7)	0.0194 (6)	0.0013 (6)	0.0055 (6)	0.0008 (6)
C6	0.0245 (8)	0.0194 (7)	0.0208 (6)	0.0052 (6)	0.0032 (6)	−0.0023 (5)
C7	0.0182 (7)	0.0179 (7)	0.0188 (6)	0.0029 (5)	−0.0006 (5)	−0.0013 (5)
C8	0.0209 (7)	0.0156 (7)	0.0211 (7)	0.0039 (5)	0.0000 (5)	−0.0026 (5)
C9	0.0193 (7)	0.0168 (7)	0.0196 (6)	0.0019 (5)	−0.0013 (5)	−0.0003 (5)
C10	0.0271 (8)	0.0153 (7)	0.0241 (7)	0.0006 (5)	−0.0006 (6)	−0.0016 (5)
C11	0.0293 (8)	0.0190 (7)	0.0249 (7)	−0.0038 (6)	0.0007 (6)	0.0018 (6)
C12	0.0228 (8)	0.0263 (8)	0.0238 (7)	−0.0020 (6)	0.0045 (6)	−0.0004 (6)
C13	0.0207 (7)	0.0198 (7)	0.0229 (7)	0.0005 (5)	0.0023 (5)	−0.0039 (5)
C14	0.0178 (7)	0.0172 (7)	0.0182 (6)	0.0008 (5)	−0.0003 (5)	−0.0017 (5)
C15	0.0197 (7)	0.0173 (7)	0.0220 (7)	0.0012 (5)	0.0033 (5)	−0.0005 (5)
C17	0.0243 (8)	0.0173 (7)	0.0269 (7)	0.0010 (6)	0.0047 (6)	−0.0062 (6)

### 1-Anthracen-9-yl-N-methylmethanimine oxide Geometric parameters (Å, º)

**Table:**

O16—N16	1.2932 (15)	C8—H8	0.9500
N16—C15	1.2975 (18)	C8—C9	1.3924 (19)
N16—C17	1.4785 (16)	C9—C10	1.4324 (19)
C1—C2	1.4108 (19)	C9—C14	1.4381 (18)
C1—C14	1.4172 (18)	C10—H10	0.9500
C1—C15	1.4757 (18)	C10—C11	1.360 (2)
C2—C3	1.4332 (18)	C11—H11	0.9500
C2—C7	1.4396 (18)	C11—C12	1.4229 (19)
C3—H3	0.9500	C12—H12	0.9500
C3—C4	1.3631 (19)	C12—C13	1.3618 (19)
C4—H4	0.9500	C13—H13	0.9500
C4—C5	1.4217 (19)	C13—C14	1.4323 (19)
C5—H5	0.9500	C15—H15	0.9500
C5—C6	1.356 (2)	C17—H17A	0.9800
C6—H6	0.9500	C17—H17B	0.9800
C6—C7	1.4293 (19)	C17—H17C	0.9800
C7—C8	1.3968 (18)		
			
O16—N16—C15	125.30 (12)	C8—C9—C10	121.67 (12)
O16—N16—C17	114.96 (11)	C8—C9—C14	119.50 (12)
C15—N16—C17	119.73 (12)	C10—C9—C14	118.84 (12)
C2—C1—C14	120.37 (11)	C9—C10—H10	119.4
C2—C1—C15	122.40 (12)	C11—C10—C9	121.13 (13)
C14—C1—C15	117.05 (11)	C11—C10—H10	119.4
C1—C2—C3	122.93 (11)	C10—C11—H11	119.9
C1—C2—C7	119.48 (12)	C10—C11—C12	120.11 (13)
C3—C2—C7	117.57 (12)	C12—C11—H11	119.9
C2—C3—H3	119.4	C11—C12—H12	119.6
C4—C3—C2	121.17 (12)	C13—C12—C11	120.74 (13)
C4—C3—H3	119.4	C13—C12—H12	119.6
C3—C4—H4	119.6	C12—C13—H13	119.4
C3—C4—C5	120.90 (13)	C12—C13—C14	121.18 (13)
C5—C4—H4	119.6	C14—C13—H13	119.4
C4—C5—H5	120.0	C1—C14—C9	119.45 (12)
C6—C5—C4	119.91 (13)	C1—C14—C13	122.54 (12)
C6—C5—H5	120.0	C13—C14—C9	118.00 (12)
C5—C6—H6	119.4	N16—C15—C1	125.01 (13)
C5—C6—C7	121.29 (12)	N16—C15—H15	117.5
C7—C6—H6	119.4	C1—C15—H15	117.5
C6—C7—C2	119.07 (12)	N16—C17—H17A	109.5
C8—C7—C2	119.44 (12)	N16—C17—H17B	109.5
C8—C7—C6	121.49 (12)	N16—C17—H17C	109.5
C7—C8—H8	119.1	H17A—C17—H17B	109.5
C9—C8—C7	121.75 (12)	H17A—C17—H17C	109.5
C9—C8—H8	119.1	H17B—C17—H17C	109.5
			
O16—N16—C15—C1	0.9 (2)	C8—C9—C10—C11	−179.17 (13)
C1—C2—C3—C4	−178.89 (12)	C8—C9—C14—C1	0.08 (19)
C1—C2—C7—C6	178.96 (12)	C8—C9—C14—C13	179.06 (12)
C1—C2—C7—C8	−1.51 (19)	C9—C10—C11—C12	−0.4 (2)
C2—C1—C14—C9	−0.90 (19)	C10—C9—C14—C1	−179.40 (12)
C2—C1—C14—C13	−179.83 (12)	C10—C9—C14—C13	−0.42 (19)
C2—C1—C15—N16	56.39 (19)	C10—C11—C12—C13	0.6 (2)
C2—C3—C4—C5	−0.7 (2)	C11—C12—C13—C14	−0.7 (2)
C2—C7—C8—C9	0.7 (2)	C12—C13—C14—C1	179.59 (13)
C3—C2—C7—C6	−2.96 (19)	C12—C13—C14—C9	0.6 (2)
C3—C2—C7—C8	176.56 (12)	C14—C1—C2—C3	−176.36 (12)
C3—C4—C5—C6	−1.8 (2)	C14—C1—C2—C7	1.61 (19)
C4—C5—C6—C7	1.9 (2)	C14—C1—C15—N16	−128.50 (14)
C5—C6—C7—C2	0.5 (2)	C14—C9—C10—C11	0.3 (2)
C5—C6—C7—C8	−179.00 (13)	C15—C1—C2—C3	−1.4 (2)
C6—C7—C8—C9	−179.77 (12)	C15—C1—C2—C7	176.56 (12)
C7—C2—C3—C4	3.1 (2)	C15—C1—C14—C9	−176.11 (11)
C7—C8—C9—C10	179.46 (12)	C15—C1—C14—C13	4.96 (19)
C7—C8—C9—C14	0.0 (2)	C17—N16—C15—C1	179.78 (12)

### 1-Anthracen-9-yl-N-methylmethanimine oxide Hydrogen-bond geometry (Å, º)

**Table:**

*D*—H···*A*	*D*—H	H···*A*	*D*···*A*	*D*—H···*A*
C15—H15···O16^i^	0.95	2.24	3.1267 (18)	154
C17—H17*B*···O16^i^	0.98	2.28	3.2037 (18)	157

Symmetry code: (i) *x*+1, *y*, *z*.
