# Supplementary material for: Crystal structure and Hirshfeld surface analysis, crystal voids, inter­action energy calculations and energy frameworks of C-anthracen-9-yl-N-methyl aldo­nitrone
Source: Acta Crystallogr E Crystallogr Commun. 2026 Jan 29;82(Pt 2):221–6. doi: 10.1107/S2056989026000599 (PMC12874247; doi:10.1107/S2056989026000599)

checkCIF/PLATON report

Structure factors have been supplied for datablock(s) lasri14

THIS REPORT IS FOR GUIDANCE ONLY. IF USED AS PART OF A REVIEW PROCEDURE FOR PUBLICATION, IT SHOULD NOT REPLACE THE EXPERTISE OF AN EXPERIENCED CRYSTALLOGRAPHIC REFEREE.

No syntax errors found. CIF dictionary Interpreting this report

Datablock: lasri14

Bond precision:	C-C = 0.0019 A		Wavelength=0.71073
Cell:	a=4.89615(14)	b=16.6590(5)	c=14.2008(4)
	alpha=90	beta=91.240(3)	gamma=90
Temperature:	100 K		

	Calculated	Reported
Volume	1158.02(6)	1158.02(6)
Space group	P 21/n	P 1 21/n 1
Hall group	-P 2yn	-P 2yn
Moiety formula	C16 H13 N O	C16 H13 N O
Sum formula	C16 H13 N O	C16 H13 N O
Mr	235.27	235.27
Dx,g cm-3	1.349	1.349
Z	4	4
Mu (mm-1)	0.084	0.084
F000	496.0	496.0
F000'	496.20	
h,k,lmax	6,22,19	6,21,19
Nref	3091	2788
Tmin,Tmax	0.998,0.999	0.714,1.000
Tmin'	0.978	

Correction method= # Reported T Limits: Tmin=0.714 Tmax=1.000
AbsCorr = MULTI-SCAN

Data completeness= 0.902 Theta(max)= 28.990

R(reflections)= 0.0432(1992) wR2(reflections)= 0.1243(2788)

S = 1.053 Npar= 164

The following ALERTS were generated. Each ALERT has the format
test-name_ALERT_alert-type_alert-level.
Click on the hyperlinks for more details of the test.

Alert level G

```
PLAT802_ALERT_4_G CIF Input Record(s) with more than 80 Characters          7 Info
PLAT912_ALERT_4_G Missing # of FCF Reflections Above STh/L=  0.600        291 Note
PLAT969_ALERT_5_G The 'Henn et al.' R-Factor-gap value ...........      3.390 Note
              Predicted wR2: Based on SigI**2  3.67 or SHELX Weight 11.81
PLAT978_ALERT_2_G Number C-C Bonds with Positive Residual Density.         15 Info
```

---

0 **ALERT level A** = Most likely a serious problem - resolve or explain
0 **ALERT level B** = A potentially serious problem, consider carefully
0 **ALERT level C** = Check. Ensure it is not caused by an omission or oversight
4 **ALERT level G** = General information/check it is not something unexpected

0 ALERT type 1 CIF construction/syntax error, inconsistent or missing data
1 ALERT type 2 Indicator that the structure model may be wrong or deficient
0 ALERT type 3 Indicator that the structure quality may be low
2 ALERT type 4 Improvement, methodology, query or suggestion
1 ALERT type 5 Informative message, check

Publication of your CIF

You should attempt to resolve as many as possible of the alerts in all categories. Often the minor alerts point to easily fixed oversights, errors and omissions in your CIF or refinement strategy, so attention to these fine details can be worthwhile. In order to resolve some of the more serious problems it may be necessary to carry out additional measurements or structure refinements. However, the nature of your study may justify the reported deviations from journal submission requirements and the more serious of these should be commented upon in the discussion or experimental section of a paper or in the "special_details" fields of the CIF. *checkCIF* was carefully designed to identify outliers and unusual parameters, but every test has its limitations and alerts that are not important in a particular case may appear. Conversely, the absence of alerts does not guarantee there are no aspects of the results needing attention. It is up to the individual to critically assess their own results and, if necessary, seek expert advice.

If you wish to submit your CIF for publication in Acta Crystallographica Section C or E, you should upload your CIF via the web. If you wish to submit your CIF for publication in IUCrData you should upload your CIF via the web. If your CIF is to form part of a submission to another IUCr journal, you will be asked, either during electronic submission or by the Co-editor handling your paper, to upload your CIF via our web site.

PLATON version of 26/09/2025; check.def file version of 20/09/2025

Datablock lasri14 - ellipsoid plot

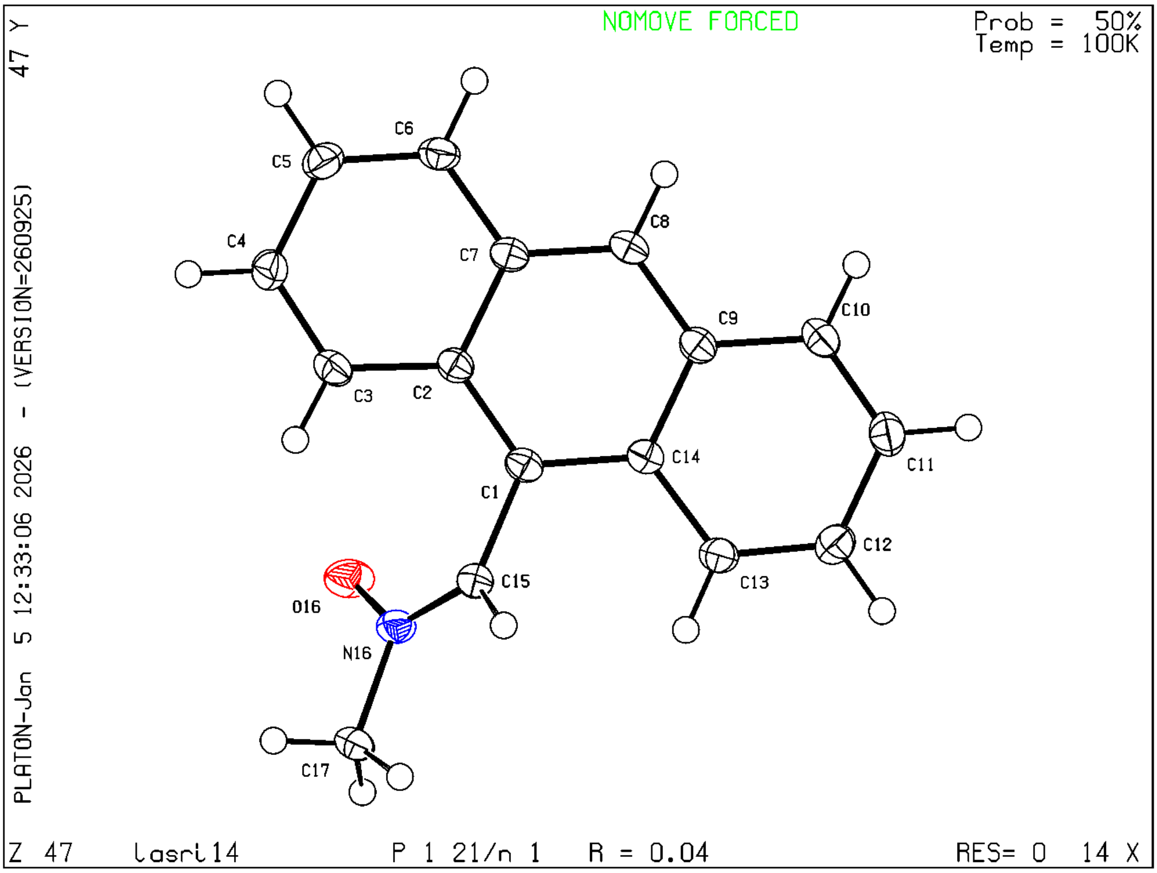

Supplement: Supplementary file 3 [file e-82-00221-sup3.pdf]
